# Childhood sexual abuse and lifetime depressive symptoms: the importance of type and timing of childhood emotional maltreatment

**DOI:** 10.1017/S003329172400268X

**Published:** 2024-11

**Authors:** Lauren M. Hutson, Rotem Dan, Aliza R. Brown, Shiba M. Esfand, Valerie Ruberto, Emily Johns, Kaylee E. Null, Kyoko Ohashi, Alaptagin Khan, Fei Du, Martin H. Teicher, Diego A. Pizzagalli

**Affiliations:** 1 Center for Depression, Anxiety and Stress Research, McLean Hospital, Belmont, MA 02478, USA; 2 Department of Psychiatry, Harvard Medical School, Boston, MA 02115, USA; 3 Developmental Biopsychiatry Research Program, McLean Hospital, Belmont, MA 02478, USA; 4 McLean Imaging Center, McLean Hospital, Belmont, MA 02478, USA

**Keywords:** adolescence, child sexual abuse, depression, life stress, machine learning

## Abstract

**Background:**

Childhood sexual abuse (CSA) and emotional maltreatment are salient risk factors for the development of major depressive disorder (MDD) in women. However, the type- and timing-specific effects of emotional maltreatment experienced during adolescence on future depressive symptomatology in women with CSA have not been explored. The goal of this study was to fill this gap.

**Methods:**

In total, 203 women (ages 20–32) with current depressive symptoms and CSA (MDD/CSA), remitted depressive symptoms and CSA (rMDD/CSA), and current depressive symptoms without CSA (MDD/no CSA) were recruited from the community and completed self-report measures. Depressive symptoms were assessed using the Beck Depression Inventory (BDI-II) and a detailed maltreatment history was collected using the Maltreatment and Abuse Chronology of Exposure (MACE). Differences in maltreatment exposure characteristics, including multiplicity and severity of maltreatment, as well as the chronologies of emotional maltreatment subtypes were compared among groups. A random forest machine-learning algorithm was utilized to assess the impact of exposure to emotional maltreatment subtypes at specific ages on current depressive symptoms.

**Results:**

MDD/CSA women reported greater prevalence and severity of emotional maltreatment relative to rMDD/CSA and MDD/no CSA women [*F*_(2,196)_ = 9.33, *p* < 0.001], specifically from ages 12 to 18. The strongest predictor of current depressive symptoms was parental verbal abuse at age 18 for both MDD/CSA women (variable importance [VI] = 1.08, *p* = 0.006) and MDD/no CSA women (VI = 0.68, *p* = 0.004).

**Conclusions:**

Targeting emotional maltreatment during late adolescence might prove beneficial for future intervention efforts for MDD following CSA.

## Introduction

Severe childhood adversity accounts for a large portion of psychiatric illness and increases the risk for major depressive disorder (MDD) (Green et al., 2010). Individuals who develop MDD following childhood maltreatment endure a more pernicious illness course characterized by longer chronicity, recurrent episodes, and unfavorable treatment outcomes (Garcia-Toro et al., 2013; Nanni, Uher, & Danese, 2012; Wiersma et al., 2009; Zlotnick, Mattia, & Zimmerman, 2001).

Recent models posit that specific maltreatment subtypes occurring during certain developmental windows have uniquely potent impacts on psychopathological outcomes (McLaughlin & Sheridan, 2016; Schaefer, Cheng, & Dunn, 2022). These type- and timing-specific effects are corroborated by research on mechanisms underlying the neurobiological embedding of adversity, as critical developmental windows have been identified during which distinct types of maltreatment maximally impact brain development (Pechtel, Lyons-Ruth, Anderson, & Teicher, 2014; Zhu et al., 2019). Examining the developmental trajectory of maltreatment exposure and identifying these critical exposure periods can also increase our understanding of resilience to future psychopathology. Moreover, identifying ages at which certain adverse experiences are most strongly associated with future depression can have important implications for more targeted and timely prevention and intervention efforts.

Among the different subtypes of maltreatment, childhood sexual abuse (CSA) has emerged as a salient predictor of MDD, with over 60% of adults meeting diagnostic criteria following CSA (Li, D'Arcy, & Meng, 2016; Noll, 2021; Teicher, Samson, Polcari, & Andersen, 2009). Moreover, women who experienced CSA are more likely to report additional childhood maltreatment subtypes relative to women who did not experience CSA (Lacelle, Hébert, Lavoie, Vitaro, & Tremblay, 2012). This finding is of clinical importance since exposure to multiple concurrent types of childhood maltreatment, particularly in adolescence, has been associated with greater risk, severity, and chronicity of subsequent depression (Lumley & Harkness, 2007; Negele, Kaufhold, Kallenbach, & Leuzinger-Bohleber, 2015; Widom, DuMont, & Czaja, 2007). CSA was particularly associated with emotional maltreatment from both parents and peers (Bailey, Baker, McElduff, & Kavanagh, 2016; Kennedy, Font, Haag, & Noll, 2022; Tremblay-Perreault & Hébert, 2020). Childhood emotional maltreatment encapsulates both threat (e.g. the child is the target of verbal threats from parents or experiences bullying from peers) and/or deprivation (e.g. parents are emotionally unavailable or do not serve as a source of strength and support to the child) experiences, both of which adversely impact development (McLaughlin & Sheridan, 2016; Teicher & Parigger, 2015). Like CSA, exposure to emotional maltreatment subtypes has been strongly associated with the development of future depression (Radell, Abo Hamza, Daghustani, Perveen, & Moustafa, 2021; Steine et al., 2017).

Childhood emotional maltreatment is the most common adverse childhood experience, and its incidence in the US has increased over time; however, its effects have historically been understudied in comparison to sexual and physical abuse (Swedo et al., 2023; Trickett, Mennen, Kim, & Sang, 2009). Furthermore, the type- and timing-specific effects of emotional maltreatment subtypes on future depressive symptomatology, specifically in women with CSA, have not been investigated. Given the incidence of childhood emotional maltreatment endorsed by women with CSA, as well as the demonstrated relationship between CSA and emotional maltreatment on future depression, it is important to identify the critical ages during which emotional maltreatment most strongly predicts depressive outcomes in women with CSA.

The present study aimed to investigate the relationship between experiences of childhood emotional maltreatment and CSA on subsequent depressive symptoms in women. First, we examined differences in multiplicity of maltreatment, severity of overall maltreatment, and exposure to distinct emotional maltreatment subtypes (parental verbal abuse, parental non-verbal emotional abuse, peer verbal abuse, and emotional neglect). We predicted that women with depressive symptoms (current or past) who experienced CSA would report greater severity of overall maltreatment characteristics (multiplicity, sum, and duration of maltreatment) as well as greater severity of emotional maltreatment, relative to women with current depressive symptoms without CSA. Second, we characterized the developmental time course of exposure to emotional maltreatment subtypes for women with depressive symptoms (current or past) and with or without CSA. Last, machine learning predictive modeling was used to identify the most important emotional maltreatment subtypes and ages for predicting future depressive symptoms in women with current depressive symptoms with and without CSA.

## Materials and methods

### Sample and study design

Women were recruited in the context of a larger neuroimaging project investigating the functional and molecular effects of CSA between the ages of 11–18 years on depression (R01 MH095809). Women were recruited from the greater Boston area primarily through advertisements on social media platforms and local college job boards. The data for the present analyses came from participants who completed the first stage of the larger neuroimaging project. Participants first completed a self-report survey that included the Beck Depression Inventory-II (BDI-II) (Beck, Steer, & Brown, 1996), as well as demographic and other information about their current and past medical and psychiatric history (see online Supplemental Methods for a full list of inclusion and exclusion criteria). Next, eligible participants completed the 75-item Maltreatment and Abuse Chronology of Exposure (MACE) (Teicher & Parigger, 2015) to obtain a history of childhood maltreatment. Self-report survey and MACE data were collected using REDCap electronic data capture tools hosted at Massachusetts General Brigham (MGB) (Harris et al., 2009, 2019). Prior to enrollment, participants provided electronic informed consent. The study was approved by and conducted in accordance with the MGB and McLean Hospital Institutional Review Board (Protocol Number: 2020P001470).

Based on the BDI-II, MACE, and phone screening, participants were categorized into three groups: (i) current depressive symptoms and CSA between the ages of 11 and 18 years (MDD/CSA), (ii) past depression without current depressive symptoms, and CSA between the ages of 11 and 18 years (rMDD/CSA), and (iii) current depressive symptoms without a history of CSA (MDD/no CSA).

### Current and past depressive symptomatology

The BDI-II was used to assess depressive symptoms over the past 2 weeks. Although this instrument has 21 questions, the suicidality question was removed due to the online nature of the questionnaire, so total scores were computed by summing the remaining 20 questions. Participants' current and past depressive symptoms were confirmed in a 30-minute phone screening conducted by trained clinical research assistants at McLean Hospital. Questions regarding current and past depressive symptomatology closely mirrored the MDD module of the mood disorders section of the research version of the Structured Clinical Interview for DSM-5 (SCID-5-RV) (First, Williams, Karg, & Spitzer, 2015).

Participants with current depressive symptoms endorsed at least five symptoms of MDD at a clinically significant level in the past 2 weeks and had a BDI score ⩾14. Participants with past depressive symptoms reported no depressed mood or anhedonia in the past 2 months, in addition to either (i) one period of at least 2 months or (ii) two separate periods of at least 2 weeks in the past 5 years where the participant endorsed at least five symptoms of MDD at a clinically significant level. They also had a current BDI-II score <14. Importantly, all participants across groups were not taking psychotropic medication at the time of enrollment.

### Childhood maltreatment exposure

A detailed maltreatment history between ages 1 and 18 years was assessed using the MACE, which evaluates the severity of the following 10 maltreatment subtypes at each age: sexual abuse, parental verbal abuse, parental non-verbal emotional abuse, parental physical abuse, witnessing abuse of siblings, witnessing interparental violence, peer verbal abuse, peer physical abuse, emotional neglect, and physical neglect.

The following global measures of maltreatment were computed:

- Maltreatment multiplicity (MULT): the number of different significant subtypes of maltreatment which the participant endorsed, ranging from 0 to 9 (sexual abuse was not included in the multiplicity score to enable comparison between women with and without CSA). Each maltreatment subscale has a numerical threshold that indicates significant exposure to that specific subtype (Teicher & Parigger, 2015). For every subtype, the severity was computed by summing the number of items endorsed between ages 1 and 18 years. If the severity score reached the threshold for that subtype, it was considered a significant exposure.
- Total maltreatment severity (SUM): the sum of the severity scores on the MACE maltreatment subscales (excluding CSA) across the ages of 1–18 years, ranging from 0 to 90.
- Total maltreatment duration: the number of years (ranging from 0 to 18 years) of significant exposure to at least one type of maltreatment, excluding CSA (i.e. the number of years where MULT > 0).

### Sexual abuse assessment

The MACE includes 12 items assessing sexual abuse by parents or adults living in the house, adults living outside the house, or peers: 2 items addressing non-contact sexual abuse (e.g. inappropriate sexual comments) and 10 items addressing contact sexual abuse. For this study, the endorsement of any contact sexual abuse item between the age of 11 and 18 years was considered substantial sexual abuse exposure. This age range was selected due to the parent study's focus on the neurodevelopmental impact of maltreatment during adolescence. Note that the MACE's original criteria for significant sexual abuse slightly differ and required endorsement of two items from a smaller subset of questions (Teicher & Parigger, 2015) (see online Supplemental Methods).

Duration of sexual abuse was defined as the number of years in which at least one contact sexual abuse item was endorsed between ages 11 and 18. Participants reporting any contact sexual abuse prior to the age of 11 were excluded from analyses.

### Chronologies of emotional maltreatment

Chronologies of emotional maltreatment were derived using the MACE severity scores for each age and subtype: parental verbal abuse (PVA), parental non-verbal emotional abuse (NVEA), peer verbal abuse (PEERVA), and emotional neglect (EN). Chronology of total emotional maltreatment severity was created by summing the severity scores for the four subtypes at each age.

### Statistical analyses

#### Sexual abuse characteristics

Differences between MDD/CSA and rMDD/CSA women with respect to number of CSA perpetrators and duration of CSA was endorsed were analyzed using independent-samples *t* tests. Differences between these two groups with respect to prevalence of CSA perpetrator relationships (i.e. adult in the house, adult outside the house, or peer) were analyzed using χ^2^ tests.

#### Between-group differences in maltreatment exposure

Analyses were conducted using IBM SPSS Statistics (Version 28.0.0.0) (SPSS Inc., Chicago, IL, USA) and R. Differences in the global maltreatment measures were examined using one-way ANCOVA models implemented by a GLM with group as a between-subject factor. Differences in the prevalences of maltreatment subtypes were evaluated using χ^2^ tests. Differences in total emotional maltreatment severity were evaluated using a two-way mixed ANCOVA with group as a between-subject factor and emotional maltreatment (PVA, NVEA, PEERVA, and EN) as a within-subject factor. Differences in emotional maltreatment severity at each age between 1 and 18 years old were evaluated using a two-way mixed ANCOVA with group as a between-subject factor and age of maltreatment as a within-subject factor. Current age, race, ethnicity, and education were included as covariates.

#### Random forest regression with conditional interference trees

Random forest regression with conditional interference trees (‘cforest’ in R package party) (Strobl, Boulesteix, Zeileis, & Hothorn, 2007) was used to examine the importance of type and timing of emotional maltreatment for current depressive symptom severity in MDD/no CSA and MDD/CSA women. This machine learning strategy is robust for detecting important predictors from a large number of predictors (Breiman, 2001; Liaw & Wiener, 2002; Svetnik et al., 2003). It has also demonstrated resistance to collinearity, which is important given the substantial collinearity in the severity of maltreatment exposure at adjacent ages (Breiman, 2001). This approach has previously been used in similar analyses of sensitive periods in which maltreatment maximally predicted psychopathology and neurodevelopmental disruption (Gokten & Uyulan, 2021; Herzog et al., 2020; Schalinski et al., 2016, 2019; Zhu et al., 2019). This analysis was conducted using in-house software written in R (by M. H. T).

PVA, NVEA, PEERVA, and EN at ages 1–18 were used as predictors. Additional MACE maltreatment subtypes (e.g. physical abuse) were not used as predictors in this model since this investigation specifically focused on emotional maltreatment. Current age, race, ethnicity, and education were accounted for as covariates. Importance was defined as the mean increase in the mean square error of the overall model fit following permutation of each independent variable. Significance was assessed using a non-parametric test that treats the array of predictors as a composite and yields a measure that controls for multiple comparisons.

Relative variable importance (VI) values for each predictor were derived to examine sensitivity by type and timing. The null hypothesis was that the VI of global MACE measures was greater than the VI of exposure to a specific emotional maltreatment subtype at a specific age. This hypothesis would be rejected if the VI of exposure to a specific maltreatment subtype surpassed the VI of both MACE MULT and SUM.

## Results

### Sample characteristics

Between March 2020 and July 2023, *N* = 3364 women completed the self-report recruitment survey. Of these respondents, 1081 were invited to complete the phone screening to further assess eligibility, and 567 women completed this assessment. In total, 247 women were eligible on the phone screening and were sent the MACE to gather a detailed maltreatment history, and 234 of these women completed the MACE. Seventeen women were excluded for endorsing CSA before age 11, and three MDD/no CSA women were excluded for endorsing witnessing CSA of their sibling. Five rMDD women were excluded for not endorsing contact CSA. Finally, six women were excluded for incomplete BDI-II scores. A study enrollment chart and detailed parent study exclusion criteria can be found in the online Supplemental Methods. The final analyzed sample comprised 203 women: 69 MDD/CSA, 21 rMDD/CSA, and 113 MDD/no CSA (Table 1). Groups were matched for age, race, ethnicity, and highest level of education completed.

**Table 1.** Demographic and clinical characteristics of the sample

	MDD/CSA	rMDD/CSA	MDD/no CSA		
	*N* = 69	*N* = 21	*N* = 113		
Demographics				*F*	*p*
Age, years, mean ± s.d. (range)	24.45 ± 3.08 (20–31)	24.28 ± 3.92 (20–32)	24.63 ± 3.45 (20–32)	1.238	0.292
Race				χ^2^	*p*
Asian, *n* (%)	11 (18.3)	2 (11.1)	22 (23.4)	13.610	0.093
Black, *n* (%)	4 (6.7)	2 (11.1)	14 (14.7)		
White, *n* (%)	34 (56.7)	10 (55.6)	53 (56.4)		
More than one race, *n* (%)	10 (16.7)	4 (22.2)	3 (3.2)		
Ethnicity				χ^2^	*p*
Hispanic, *n* (%)	8 (13.3)	2 (11.1)	11 (11.7)	0.145	0.930
Education				χ^2^	*p*
High school, *n* (%)	1 (1.7)	0	3 (3.2)	7.491	0.278
Some college, *n* (%)	23 (38.3)	7 (38.9)	27 (28.7)		
4-year college, *n* (%)	18 (30.0)	4 (22.2)	27 (28.7)		
Graduate or professional school, *n* (%)	18 (30.0)	7 (38.9)	37 (39.5)		
Depressive symptoms				*F*	*p*
BDI-II, mean ± s.d.	32.05 ± 8.85	5.17 ± 3.96	27.83 ± 7.96	94.557	< 0.001

BDI, Beck Depression Inventory II.

MDD/CSA participants reported more severe current depressive symptoms (BDI) than MDD/no CSA women (Sidak; *p* = 0.007).

### Sexual abuse characteristics

Of the participants reporting CSA between 11 and 18 years old (MDD/CSA and rMDD/CSA; *N* = 90), 61.1% of women (*N* = 55) reported CSA perpetrated by a peer their own age or older, 56.7% of women (*N* = 51) reported CSA perpetrated by an adult not living in the house, and 11.1% of women (*N* = 10) reported CSA perpetrated by parents/adults living in the house. Notably, all women who endorsed CSA from an adult or older individual living in their home were in the MDD/CSA group. Additionally, MDD/CSA women more commonly reported CSA from a peer relative to rMDD/CSA women (76.7 and 23.3%, respectively) (χ^2^ = 6.11; *p* = 0.013).

In total, 24.4% of women (*N* = 22) reported CSA from multiple perpetrators during this age range. MDD/CSA women endorsed having experienced CSA from a greater number of perpetrators compared to rMDD/CSA women (*t*[85.047] = 3.82, *p* < 0.001). Finally, 68% of women (*N* = 53) reported CSA at more than one age. The average duration of CSA was 2.51 ± 1.62 years for MDD/CSA women and 1.86 ± 1.01 years for rMDD/CSA women, which was not significantly different between groups (*t*[88] = 1.73, *p* = 0.087). A table of sexual abuse characteristics can be found in online Supplemental Table S1.

### Global measures of childhood maltreatment between 1 and 18 years old

#### Multiplicity of maltreatment (MULT)

**Figure 1.**
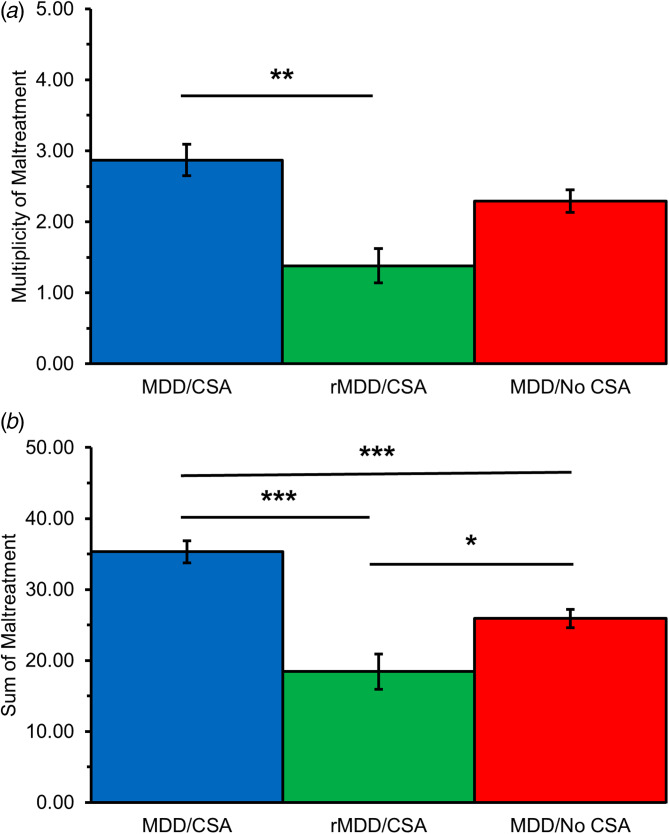
Between-group differences in MACE global measures of maltreatment. **(A)** MDD/CSA women reported greater multiplicity of maltreatment than rMDD/CSA women. **(B)** MDD/CSA women reported greater severity of maltreatment than rMDD/CSA women and MDD/No CSA women. MDD/No CSA women also reported greater severity of maltreatment than rMDD/CSA women. Significance: *p < .05, **p < .01, ***p < .001.

Significant differences in multiplicity of maltreatment between the ages of 1 and 18 years (excluding CSA) were reported among groups (*F*_[2,196]_ = 5.60, *p* = 0.004, *η_p_^2^* = 0.056) (Fig. 1a). Specifically, MDD/CSA women reported greater multiplicity of maltreatment relative to rMDD/CSA women (Sidak; *p* = 0.003; 95% CI [3.750–2.399]). Education was a significant covariate (*F*_[1,191]_ = 9.91, *p* = 0.002, *η_p_^2^* = 0.049).

#### Severity of maltreatment (SUM)

Significant differences in total severity of maltreatment between 1 and 18 years old emerged across groups (*F*_[2,191]_ = 15.90, *p* < 0.001, *η_p_^2^* = 0.143) (Fig. 1b). MDD/CSA women reported greater severity relative to rMDD/CSA women (Sidak; *p* < 0.001; 95% CI [8.462–23.995]) and MDD/no CSA women (Sidak; *p* < 0.001; 95% CI [3.599–13.404]). In addition, MDD/no CSA women reported greater severity of maltreatment relative to rMDD/CSA women (Sidak; *p* = 0.031; 95% CI [0.164–15.291]). Race and education were significant covariates (*F*_[1,196]_ = 4.52, *p* = 0.035, *η_p_^2^* = 0.025 and *F*_[1,191]_ = 8.34, *p* = 0.004, *η_p_^2^* = 0.042, respectively).

#### Duration of maltreatment

There were no significant between-group differences in duration of maltreatment. Race and education were significant covariates (*F*_[1,196]_ = 4.10, *p* = 0.044, *η_p_^2^* = 0.050 and *F*_[1,196]_ = 10.18, *p* = 0.002, *η_p_^2^* = 0.051, respectively).

### Maltreatment subtype prevalence

Table 2 presents the prevalence of significant exposure to maltreatment subtypes for each group. MDD/CSA women reported higher prevalence of significant exposure to emotional maltreatment subtypes, including PVA (χ^2^ = 15.71; *p* < 0.001), PEERVA (χ^2^ = 8.91; *p* = 0.012), EN (χ^2^ = 7.30; *p* = 0.026), and NVEA (χ^2^ = 7.20; *p* = 0.027), in addition to higher prevalence of peer physical abuse (χ^2^ = 10.18; *p* = 0.006) compared to MDD/no CSA and rMDD/CSA women.

**Table 2.** MACE maltreatment subtype prevalence between 1 and 18 years old

	MDD/CSA	rMDD/CSA	MDD/no CSA	Total		
	*N* = 69	*N* = 21	*N* = 113	*N* = 203	χ^2^	*p*
MACE maltreatment subtype, *n* (%)						
Emotional neglect	55 (79.7)	12 (51.7)	70 (61.9)	137 (67.5)	7.30	0.026
Parental verbal abuse	44 (63.8)	4 (19.0)	47 (41.6)	95 (46.8)	15.71	< 0.001
Physical neglect	36 (52.2)	7 (33.3)	47 (41.6)	90 (44.3)	3.09	0.213
Peer verbal abuse	38 (55.1)	8 (38.1)	37 (32.7)	83 (41.0)	8.91	0.012
Witnessing violence toward siblings	22 (31.9)	3 (14.3)	27 (23.9)	52 (25.6)	3.01	0.222
Parental non-verbal emotional abuse	25 (36.2)	3 (14.3)	23 (20.4)	51 (25.1)	7.20	0.027
Witnessing interparental violence	18 (26.1)	1 (4.8)	18 (15.9)	37 (18.2)	5.82	0.055
Parental physical violence	14 (20.3)	2 (9.5)	17 (15.0)	33 (16.3)	1.65	0.439
Peer physical abuse	12 (17.4)	2 (9.5)	4 (3.5)	18 (8.9)	10.18	0.006

Comparisons of significant exposure to nine MACE maltreatment subtypes between groups. MDD/CSA women were more likely to report significant exposure to emotional neglect, parental verbal abuse, peer verbal abuse, parental non-verbal emotional abuse, and peer physical abuse than rMDD/CSA and MDD/no CSA women. Significant exposure was defined as severity meeting or exceeding the severity threshold for each maltreatment subtype on the MACE. MACE, Maltreatment and Abuse Chronology of Exposure.

### Women with current depressive symptoms and CSA report greater severity of emotional maltreatment

There were significant between-group differences in emotional maltreatment severity (*F*_[2,196]_ = 17.60, *p* < 0.001, *η_p_^2^* = 0.152). Across emotional maltreatment subtypes (PVA, NVEA, PEERVA, and EN), MDD/CSA women reported greater emotional maltreatment severity compared to MDD/no CSA women (Sidak; *p* < 0.001; 95% CI [0.653–2.147]) and rMDD/CSA women (Sidak; *p* < 0.001; 95% [1.440–3.853]). MDD/no CSA women also reported greater emotional maltreatment severity compared to rMDD/CSA women (Sidak; *p* = 0.029; 95% CI [0.095–2.397]). Education was a significant covariate (*F*_[1,196]_ = 9.98, *p* = 0.002, *η_p_^2^* = 0.048).

**Figure 2.**
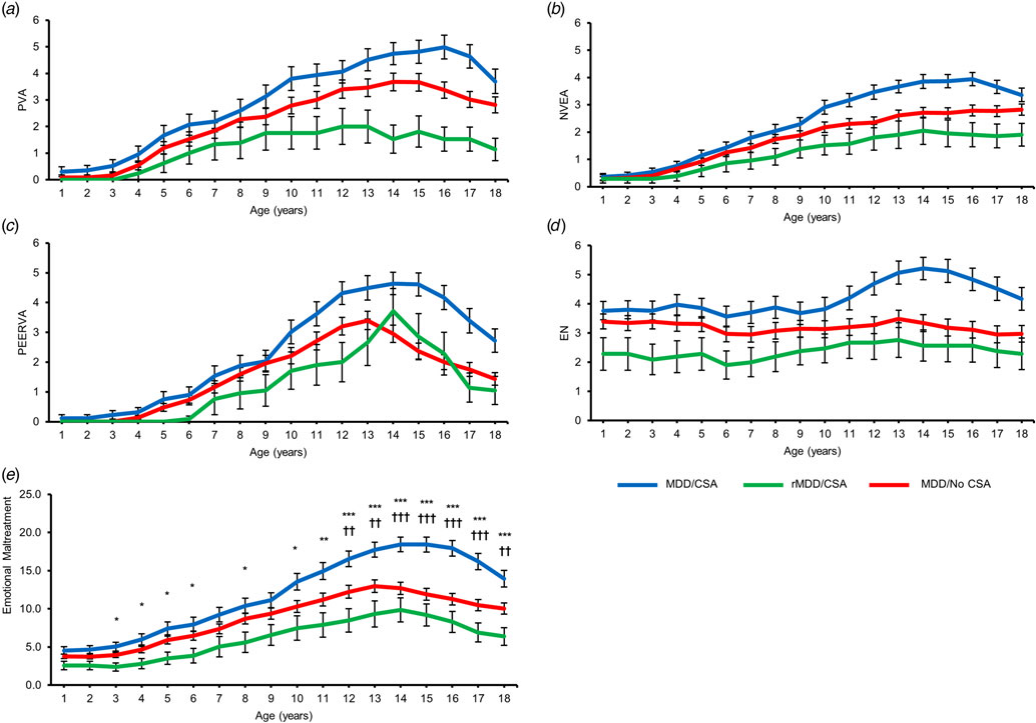
Chronology of emotional maltreatment (PVA, NVEA, PEERVA, and EN) severity and age-specific severity differences. **(A)** Severity of exposure to parental verbal abuse (PVA) between 1-18 years old. **(B)** Severity of exposure to parental non-verbal emotional abuse (NVEA) between 1-18 years old. **(C)** Severity of exposure to peer verbal abuse (PEERVA) between 1-18 years old. **(D)** Severity of exposure to emotional neglect (EN) between 1-18 years old. **(E)** MDD/CSA women reported greater severity of total emotional maltreatment than rMDD/CSA women (Sidak; p < .001) and MDD/No CSA women (Sidak; *p* = .007). MDD/CSA women specifically reported greater severity of emotional maltreatment than MDD/No CSA and rMDD/CSA women from ages 12 to 18 years old. Significance for MDD/CSA and rMDD/CSA comparisons: *p < .05, **p < .01, ***p < .001. Significance for MDD/CSA and MDD/No CSA comparisons: †p < .05, ††*p* < .01, †††*p* < .001. Abbreviations: PVA = Parental Verbal Abuse, NVEA = Parental Nonverbal Emotional Abuse, PEERVA = Peer Verbal Abuse, EN = Emotional Neglect.

For total emotional maltreatment, a group-by-age interaction emerged (*F*_[7.619,746.637]_ = 5.13, *p* < 0.001, *η_p_^2^* = 0.050) in addition to a significant main effect of group (*F*_[2,196]_ = 9.33, *p* < 0.001, *η_p_^2^* = 0.087) (Fig. 2e). MDD/CSA women reported greater total emotional maltreatment relative to rMDD/CSA women (Sidak; *p* < 0.001; 95% CI [2.263–9.181]) and MDD/no CSA women (Sidak; *p* = 0.007; 95% CI [0.587–4.868]). A main effect of age was also found (*F*_[3.809,746.637]_ = 2.50, *p* = 0.044, *η_p_^2^* = 0.013, Greenhouse–Geisser corrected). Sidak-corrected post-hoc tests revealed that MDD/CSA women reported greater emotional maltreatment than rMDD/CSA women at ages 3, 4, 5, 6, 8, 10, and 11 and greater emotional maltreatment than both rMDD/CSA and MDD/no CSA women from ages 12 to 18. Online Supplemental Table S1 presents the significant pairwise differences for each age.

### Type- and timing-specific effects of emotional maltreatment subtypes

**Figure 3.**
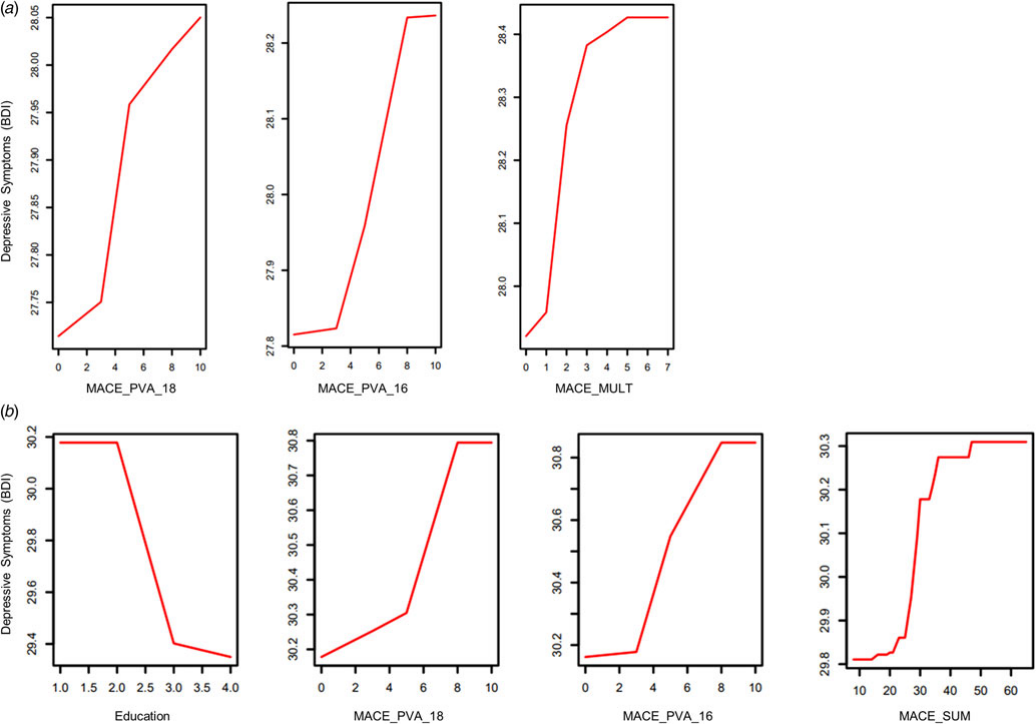
Strongest predictors of depressive symptoms in MDD/No CSA and MDD/CSA women. Dose-response curves of emotional maltreatment severity and current depressive symptoms indicating importance of type and timing of maltreatment on current depressive symptoms derived from the random forest regression with conditional interference trees. **(A)** For MDD/No CSA women, the strongest predictors of depressive symptoms were PVA at age 18 (VI = 0.68; *p* = .004), PVA at age 16 (VI = 0.56; *p* = .007), and MACE MULT (VI = 1.09; *p* = .014). Depressive symptoms increased as severity of each of these maltreatment predictors increased. **(B)** For MDD/CSA women, the strongest predictors of depressive symptoms were highest level of education completed (VI = 1.34; *p* = .005), PVA at age 18 (VI = 1.08; *p* = .006), PVA at age 16 (VI = 1.14; *p* = .009), and MACE SUM (*p* = .012). Depressive symptoms decreased as the level of education completed decreased. Depressive symptoms increased as severity of maltreatment predictors (MACE_PVA_18, MACE_PVA_16, and MACE_SUM) increased. Abbreviations: PVA = Parental Verbal Abuse; MULT = Multiplicity; SUM = Total Severity.

Figure 3 presents the dose–response curves of emotional maltreatment severity and depressive symptoms for the most important predictors identified by the random forest model. For MDD/no CSA women, the strongest predictor for depressive symptoms was parental verbal abuse at age 18 (VI = 0.68; *p* = 0.004), followed by parental verbal abuse at the age of 16 (VI = 0.56; *p* = 0.007) (Fig. 3a). Notably, this was a more important predictor than both MULT and SUM, although MULT was also a significant predictor for this group (VI = 1.09; *p* = 0.014).

For MDD/CSA women, the most important predictor for depressive symptoms was education (VI = 1.34; *p* = 0.005), with women who have completed a higher level of education reporting lower depressive symptoms (Fig. 3b). The second and third strongest predictors were parental verbal abuse at age 18 (VI = 1.08; *p* = 0.006) and at age 16 (VI = 1.14; *p* = 0.009). These were more important predictors than both MULT and SUM, although SUM was also a significant predictor for this group (VI = 1.09; *p* = 0.012).

## Discussion

The current investigation aimed to delineate the developmental trajectory of exposure to emotional maltreatment and identify at which ages certain emotional maltreatment subtypes most strongly predict future depressive symptoms in women with CSA. Our results highlight significant exposure to emotional maltreatment from both parents and peers in both early childhood and throughout adolescence among women with CSA who later develop depressive symptoms. Importantly, parental verbal abuse in late adolescence had the strongest predictive strength of future depressive symptoms in women with current depressive symptoms with and without CSA.

Women with current depressive symptoms and CSA reported greater overall severity of maltreatment compared to both women with remitted depressive symptoms and CSA and women with current depressive symptoms without CSA. Women with current depressive symptoms and CSA specifically endorsed greater severity of emotional maltreatment from both parents and peers, which persisted from early childhood into adolescence. Significant childhood maltreatment spanning several developmental periods – as demonstrated by this sample – has been found to have stronger and more negative consequences cross-diagnostically (Manly, Kim, Rogosch, & Cicchetti, 2001; Russotti et al., 2021; Thornberry, Ireland, & Smith, 2001). Our findings bolster previous research indicating that women with CSA are more likely to report a multiplicity of maltreatment and specifically heightened emotional maltreatment compared to women without history of sexual abuse (Carey, Walker, Rossouw, Seedat, & Stein, 2008; Lacelle et al., 2012). Notably, our findings add to previous literature by identifying the specific ages of heightened emotional maltreatment for women with CSA who developed later depressive symptoms.

An effect of the current depressive state emerged for both global and emotional maltreatment, where women with current depressive symptoms reported greater maltreatment. One possible explanation is mood-congruent memory bias, where women are appraising their maltreatment experiences in light of their current depressive state, which is an important consideration given this study's association between current depressive symptoms and retrospective maltreatment experiences. However, self-reports of childhood maltreatment have demonstrated consistency over time and across major depressive episodes in young women, with changes in reporting unattributable to changes in psychological symptoms (Goltermann et al., 2023; Pinto, Correia, & Maia, 2014). These findings are corroborated by a recent meta-analysis demonstrating the reliability of retrospective accounts of maltreatment compared to prospective accounts (Baldwin, Coleman, Francis, & Danese, 2024). Thus, a more likely possibility is that the impact of CSA on future depressive outcomes is worsened by exposure to emotional maltreatment and attenuated by strong relationships with both parents and peers. Although sexual abuse has been associated with non-remission from MDD and the persistence of chronic MDD (Enns & Cox, 2005; Garcia-Toro et al., 2013; Hovens, Giltay, Van Hemert, & Penninx, 2016), other studies have found that emotional maltreatment subtypes emerge as more important predictor variables for determining risk for MDD and symptom severity in women than both physical and sexual abuse (Martins, Von Werne Baes, De Carvalho Tofoli, & Juruena, 2014; Teicher, Samson, Polcari, & McGreenery, 2006). Positive familial and peer relationships have been associated with more rapid recovery as well as psychological resilience particularly following CSA (Domhardt, Münzer, Fegert, & Goldbeck, 2015; Fuller-Thomson, Lacombe-Duncan, Goodman, Fallon, & Brennenstuhl, 2020; Marriott, Hamilton-Giachritsis, & Harrop, 2014). Specifically, peer social support in adolescence has been associated with lower levels of depression following childhood emotional maltreatment (Dong, Dong, Chen, & Yang, 2023; Glickman, Choi, Lussier, Smith, & Dunn, 2021). The significant differences found here in the severity of diverse emotional maltreatment subtypes between women with current and remitted depression with CSA, particularly during adolescence, necessitate additional research on the mechanisms through which emotional maltreatment worsens depressive outcomes for women with history of CSA.

Highest level of education completed was identified as the strongest predictor of depressive symptoms in women with CSA, above emotional maltreatment at any specific age. Previous investigations of CSA and higher education have identified that the psychological consequences of CSA specifically at earlier ages of onset are associated with lower levels of educational achievement (Hardner, Wolf, & Rinfrette, 2018). Alternatively, adult women with the history of CSA have regarded their higher education as an important part of their healing process (LeBlanc, Brabant, & Forsyth, 1996). Thus, earlier psychological intervention for CSA victims may minimize or alleviate these developmental disruptions such that they can attain the potential psychological benefits of higher education. Additional research is necessary to elucidate the specific relationships between psychological development and educational outcomes in women with CSA.

The endorsement of severe parental verbal abuse by women with depressive symptoms with and without CSA may have important implications for treatment of MDD. Although parental verbal abuse has been previously suggested to confer risk for internalizing disorders and other emotional problems (Sachs-Ericsson et al., 2010) as well as neurodevelopmental abnormalities (Tomoda et al., 2011), the impact of emotional maltreatment experience on psychological outcomes is understudied compared to physical and sexual abuse. Parental support has previously been indicated as a strong predictor of psychological resilience and fewer emotional difficulties following CSA (Spaccarelli & Kim, 1995; Yancey & Hansen, 2010). However, previous research focusing on parent–child relationships among CSA survivors has focused on the impact of parental interactions and support following CSA rather than that of parental maltreatment occurring before and during these experiences. Our identification of late adolescence as a sensitivity period where parental verbal abuse is maximally associated with future depressive symptoms in women with CSA has important implications for the timing of interventions addressing parental maltreatment.

CSA and emotional maltreatment were found to substantially overlap during late adolescence. These findings highlight the importance for future research investigating how emotional maltreatment experiences from parents and peers interact and exert synergistic effects in CSA survivors, including poorer emotional regulation (Amédée, Tremblay-Perreault, Hébert, & Cyr, 2019) and impaired establishment of healthy relationships (Noll, 2021). It is additionally worth noting that, relative to rMDD/CSA women, a significantly greater number of MDD/CSA women endorsed CSA from multiple perpetrators as well as CSA perpetrated by peers. Experiencing CSA from multiple perpetrators has been previously associated with a greater number of lifetime depressive episodes (Liu, Jager-Hyman, Wagner, Alloy, & Gibb, 2012). Recent attention has been given to sexual victimization and dating violence occurring in adolescent romantic partnerships, which has been associated with both depression (Taquette & Monteiro, 2019) and revictimization in young adulthood (Manchikanti Gómez, 2011).

The present study has several limitations. First, depressive symptoms were not assessed using a structured clinical interview; thus, information regarding MDD illness course (e.g. duration, number of previous episodes) was not collected, as well as whether the participant met diagnostic criteria for current or remitted MDD. Replicating these findings using structured clinical interviews would allow investigations of the predictive strength of emotional maltreatment characteristics on other clinical features in women with CSA. Moreover, it would allow for a more comprehensive picture of the course of depressive illness, including recurrent episodes or sustained remission. Second, this study specifically focused on the impact of CSA experienced between 11 and 18 years old; however, more than half of the women reporting CSA on the initial self-report recruitment survey reported their first CSA exposure before the age of 11. Moreover, data were not collected on sexual revictimization occurring after age 18, which is associated with more severe depression (Najdowski & Ullman, 2011). Finally, this investigation only explored the impact of emotional maltreatment and CSA in women. CSA is also associated with heightened depressive symptoms in men which can be mitigated by social support (Easton, Kong, Gregas, Shen, & Shafer, 2019). Future research should investigate specific maltreatment experiences that demonstrate predictive strength for later affective psychopathology in men to inform the development of appropriate and inclusive maltreatment prevention and intervention efforts.

## Conclusions

Emotional maltreatment from parents and peers is a salient component of the maltreatment histories of women who later develop depressive symptoms, and specifically those who have experienced CSA. Our results highlight vulnerability periods in late adolescence during which parental verbal abuse maximally impacts future development of depressive symptoms in CSA victims. This sensitivity period provides evidence for type- and timing-specific models of the effects of childhood maltreatment on psychopathological development. These findings necessitate future research on the mechanisms through which psychological and other adverse health consequences can be attenuated through treatments specifically targeting the potential impact of parental relationships in women with CSA.

## Supporting information

Hutson et al. supplementary material 1 Hutson et al. supplementary material

Hutson et al. supplementary material 2 Hutson et al. supplementary material

## Supplementary material

The supplementary material for this article can be found at https://doi.org/10.1017/S003329172400268X

## References

[ref1] Amédée, L. M., Tremblay-Perreault, A., Hébert, M., & Cyr, C. (2019). Child victims of sexual abuse: Teachers’ evaluation of emotion regulation and social adaptation in school. Psychology in the Schools, 56(7), 1077–1088. 10.1002/pits.22236

[ref2] Bailey, K., Baker, A., McElduff, P., & Kavanagh, D. (2016). The influence of parental emotional neglect on assault victims seeking treatment for depressed mood and alcohol misuse: A pilot study. Journal of Clinical Medicine, 5(10), 88. 10.3390/jcm5100088 27735838 PMC5086590

[ref3] Baldwin, J. R., Coleman, O., Francis, E. R., & Danese, A. (2024). Prospective and retrospective measures of child maltreatment and their association with psychopathology: A systematic review and meta-analysis. JAMA Psychiatry, 81(8), 769–781. 10.1001/jamapsychiatry.2024.0818 38691376 PMC11063927

[ref4] Beck, A. T., Steer, R. A., & Brown, G. (1996). Beck Depression Inventory – Second Edition: Manual. San Antonio, TX: The Psychological Corporation.

[ref5] Breiman, L. (2001). Random forests. Machine Learning, 45(1), 5–32. 10.1023/A:1010933404324

[ref6] Carey, P. D., Walker, J. L., Rossouw, W., Seedat, S., & Stein, D. J. (2008). Risk indicators and psychopathology in traumatised children and adolescents with a history of sexual abuse. European Child & Adolescent Psychiatry, 17(2), 93–98. 10.1007/s00787-007-0641-0 17876504

[ref7] Domhardt, M., Münzer, A., Fegert, J. M., & Goldbeck, L. (2015). Resilience in survivors of child sexual abuse: A systematic review of the literature. Trauma, Violence, & Abuse, 16(4), 476–493. 10.1177/1524838014557288 25389279

[ref8] Dong, S., Dong, Q., Chen, H., & Yang, S. (2023). Childhood emotional neglect and adolescent depression: The role of self-compassion and friendship quality. Current Psychology, 42(28), 24451–24463. 10.1007/s12144-022-03539-4

[ref9] Easton, S. D., Kong, J., Gregas, M. C., Shen, C., & Shafer, K. (2019). Child sexual abuse and depression in late life for men: A population-based, longitudinal analysis. The Journals of Gerontology: Series B, 74(5), 842–852. 10.1093/geronb/gbx114 PMC6566324 29029215

[ref10] Enns, M. W., & Cox, B. J. (2005). Psychosocial and clinical predictors of symptom persistence vs remission in major depressive disorder. The Canadian Journal of Psychiatry, 50(12), 769–777. 10.1177/070674370505001206 16408525

[ref11] First, M., Williams, J., Karg, R., & Spitzer, R. (2015). Structured clinical interview for DSM-5 – research version *(*SCID-5 for DSM-5, research version; SCID-5–RV*)*. Arlington, VA: American Psychiatric Association.

[ref12] Fuller-Thomson, E., Lacombe-Duncan, A., Goodman, D., Fallon, B., & Brennenstuhl, S. (2020). From surviving to thriving: Factors associated with complete mental health among childhood sexual abuse survivors. Social Psychiatry and Psychiatric Epidemiology, 55(6), 735–744. 10.1007/s00127-019-01767-x 31565755

[ref13] Garcia-Toro, M., Rubio, J. M., Gili, M., Roca, M., Jin, C. J., Liu, S.-M., … Blanco, C. (2013). Persistence of chronic major depression: A national prospective study. Journal of Affective Disorders, 151(1), 306–312. 10.1016/j.jad.2013.06.013 23866303

[ref14] Glickman, E. A., Choi, K. W., Lussier, A. A., Smith, B. J., & Dunn, E. C. (2021). Childhood emotional neglect and adolescent depression: Assessing the protective role of peer social support in a longitudinal birth cohort. Frontiers in Psychiatry, 12, 681176. 10.3389/fpsyt.2021.681176 34434126 PMC8381469

[ref15] Gokten, E. S., & Uyulan, C. (2021). Prediction of the development of depression and post-traumatic stress disorder in sexually abused children using a random forest classifier. Journal of Affective Disorders, 279, 256–265. 10.1016/j.jad.2020.10.006 33074145

[ref16] Goltermann, J., Meinert, S., Hülsmann, C., Dohm, K., Grotegerd, D., Redlich, R., … Dannlowski, U. (2023). Temporal stability and state-dependence of retrospective self-reports of childhood maltreatment in healthy and depressed adults. Psychological Assessment, 35(1), 12–22. 10.1037/pas0001175 36355690

[ref17] Green, J. G., McLaughlin, K. A., Berglund, P. A., Gruber, M. J., Sampson, N. A., Zaslavsky, A. M., & Kessler, R. C. (2010). Childhood adversities and adult psychiatric disorders in the national comorbidity survey replication I: Associations with first onset of *DSM-IV* disorders. Archives of General Psychiatry, 67(2), 113. 10.1001/archgenpsychiatry.2009.186 20124111 PMC2822662

[ref18] Hardner, K., Wolf, M. R., & Rinfrette, E. S. (2018). Examining the relationship between higher educational attainment, trauma symptoms, and internalizing behaviors in child sexual abuse survivors. Child Abuse & Neglect, 86, 375–383. 10.1016/j.chiabu.2017.10.007 29074261

[ref41] Harris, P. A., Taylor, R., Minor, B. L., Elliott, V., Fernandez, M., O'Neal, L., … REDCap Consortium. (2019). The REDCap consortium: Building an international community of software platform partners. Journal of Biomedical Informatics, 95, 103208. 10.1016/j.jbi.2019.103208 31078660 PMC7254481

[ref19] Harris, P. A., Taylor, R., Thielke, R., Payne, J., Gonzalez, N., & Conde, J. G. (2009). Research electronic data capture (REDCap) – A metadata-driven methodology and workflow process for providing translational research informatics support. Journal of Biomedical Informatics, 42(2), 377–381. 10.1016/j.jbi.2008.08.010 18929686 PMC2700030

[ref20] Herzog, J. I., Thome, J., Demirakca, T., Koppe, G., Ende, G., Lis, S., … Schmahl, C. (2020). Influence of severity of type and timing of retrospectively reported childhood maltreatment on female amygdala and hippocampal volume. Scientific Reports, 10(1), Article 1. 10.1038/s41598-020-57490-0 PMC7002661 32024861

[ref21] Hovens, J. G. F. M., Giltay, E. J., Van Hemert, A. M., & Penninx, B. W. J. H. (2016). Childhood maltreatment and the course of depressive and anxiety disorders: The contribution of personality characteristics: Research article: Childhood maltreatment and mood disorders. Depression and Anxiety, 33(1), 27–34. 10.1002/da.22429 26418232

[ref22] Kennedy, R. S., Font, S. A., Haag, A.-C., & Noll, J. G. (2022). Childhood sexual abuse and exposure to peer bullying victimization. Journal of Interpersonal Violence, 37(19–20), NP18589–NP18613. 10.1177/08862605211037420 34467800 PMC11155230

[ref23] Lacelle, C., Hébert, M., Lavoie, F., Vitaro, F., & Tremblay, R. E. (2012). Child sexual abuse and women's sexual health: The contribution of CSA severity and exposure to multiple forms of childhood victimization. Journal of Child Sexual Abuse, 21(5), 571–592. 10.1080/10538712.2012.688932 22994694

[ref24] LeBlanc, J. B., Brabant, S., & Forsyth, C. J. (1996). The meaning of college for survivors of sexual abuse: Higher education and the older female college student. American Journal of Orthopsychiatry, 66(3), 468–473. 10.1037/h0080197 8827270

[ref25] Li, M., D'Arcy, C., & Meng, X. (2016). Maltreatment in childhood substantially increases the risk of adult depression and anxiety in prospective cohort studies: Systematic review, meta-analysis, and proportional attributable fractions. Psychological Medicine, 46(4), 717–730. 10.1017/S0033291715002743 26708271

[ref26] Liaw, A., & Wiener, M. (2002). Classification and regression by RandomForest. R News, 2, 18–22. https://www.scirp.org/reference/referencespapers?referenceid=2107686.

[ref27] Liu, R. T., Jager-Hyman, S., Wagner, C. A., Alloy, L. B., & Gibb, B. E. (2012). Number of childhood abuse perpetrators and the occurrence of depressive episodes in adulthood. Child Abuse & Neglect, 36(4), 323–332. 10.1016/j.chiabu.2011.11.007 22565039 PMC3359412

[ref28] Lumley, M. N., & Harkness, K. L. (2007). Specificity in the relations among childhood adversity, early maladaptive schemas, and symptom profiles in adolescent depression. Cognitive Therapy and Research, 31(5), 639–657. 10.1007/s10608-006-9100-3

[ref29] Manchikanti Gómez, A. (2011). Testing the cycle of violence hypothesis: Child abuse and adolescent dating violence as predictors of intimate partner violence in young adulthood. Youth & Society, 43(1), 171–192. 10.1177/0044118X09358313

[ref30] Manly, J. T., Kim, J. E., Rogosch, F. A., & Cicchetti, D. (2001). Dimensions of child maltreatment and children's adjustment: Contributions of developmental timing and subtype. Development and Psychopathology, 13(4), 759–782. 10.1017/S0954579401004023 11771907

[ref31] Marriott, C., Hamilton-Giachritsis, C., & Harrop, C. (2014). Factors promoting resilience following childhood sexual abuse: A structured, narrative review of the literature. Child Abuse Review, 23(1), 17–34. 10.1002/car.2258

[ref32] Martins, C. M. S., Von Werne Baes, C., De Carvalho Tofoli, S. M., & Juruena, M. F. (2014). Emotional abuse in childhood is a differential factor for the development of depression in adults. Journal of Nervous & Mental Disease, 202(11), 774–782. 10.1097/NMD.0000000000000202 25268154

[ref33] McLaughlin, K. A., & Sheridan, M. A. (2016). Beyond cumulative risk: A dimensional approach to childhood adversity. Current Directions in Psychological Science : A Journal of the American Psychological Society, 25(4), 239–245. 10.1177/0963721416655883 PMC5070918 27773969

[ref34] Najdowski, C. J., & Ullman, S. E. (2011). The effects of revictimization on coping and depression in female sexual assault victims. Journal of Traumatic Stress, 24(2), 218–221. 10.1002/jts.20610 21394789

[ref35] Nanni, V., Uher, R., & Danese, A. (2012). Childhood maltreatment predicts unfavorable course of illness and treatment outcome in depression: A meta-analysis. American Journal of Psychiatry, 169(2), 141–151. 10.1176/appi.ajp.2011.11020335 22420036

[ref36] Negele, A., Kaufhold, J., Kallenbach, L., & Leuzinger-Bohleber, M. (2015). Childhood trauma and its relation to chronic depression in adulthood. Depression Research and Treatment, 2015, 1–11. 10.1155/2015/650804 PMC4677006 26693349

[ref37] Noll, J. G. (2021). Child sexual abuse as a unique risk factor for the development of psychopathology: The compounded convergence of mechanisms. Annual Review of Clinical Psychology, 17(1), 439–464. 10.1146/annurev-clinpsy-081219-112621 PMC9301660 33472010

[ref38] Pechtel, P., Lyons-Ruth, K., Anderson, C. M., & Teicher, M. H. (2014). Sensitive periods of amygdala development: The role of maltreatment in preadolescence. NeuroImage, 97, 236–244. 10.1016/j.neuroimage.2014.04.025 24736182 PMC4258391

[ref39] Pinto, R., Correia, L., & Maia, Â (2014). Assessing the reliability of retrospective reports of adverse childhood experiences among adolescents with documented childhood maltreatment. Journal of Family Violence, 29(4), 431–438. 10.1007/s10896-014-9602-9

[ref40] Radell, M. L., Abo Hamza, E. G., Daghustani, W. H., Perveen, A., & Moustafa, A. A. (2021). The impact of different types of abuse on depression. Depression Research and Treatment, 2021, 1–12. 10.1155/2021/6654503 PMC8060108 33936814

[ref42] Russotti, J., Warmingham, J. M., Duprey, E. B., Handley, E. D., Manly, J. T., Rogosch, F. A., & Cicchetti, D. (2021). Child maltreatment and the development of psychopathology: The role of developmental timing and chronicity. Child Abuse & Neglect, 120, 105215. 10.1016/j.chiabu.2021.105215 34293550 PMC8384692

[ref43] Sachs-Ericsson, N., Gayman, M., Kendall-Tackett, K., Lloyd, D., Raines, A., Rushing, N., … Sawyer, K. (2010). The long-term impact of childhood abuse on internalizing disorders among older adults: The moderating role of self-esteem. Aging & Mental Health, 14, 489–501. 10.1080/13607860903191382 20455125

[ref44] Schaefer, J. D., Cheng, T. W., & Dunn, E. C. (2022). Sensitive periods in development and risk for psychiatric disorders and related endpoints: A systematic review of child maltreatment findings. The Lancet Psychiatry, 9(12), 978–991. 10.1016/S2215-0366(22)00362-5 36403600 PMC10443538

[ref45] Schalinski, I., Teicher, M. H., Nischk, D., Hinderer, E., Müller, O., & Rockstroh, B. (2016). Type and timing of adverse childhood experiences differentially affect severity of PTSD, dissociative and depressive symptoms in adult inpatients. BMC Psychiatry, 16(1), 295. 10.1186/s12888-016-1004-5 27543114 PMC4992284

[ref46] Schalinski, I., Breinlinger, S., Hirt, V., Teicher, M. H., Odenwald, M., & Rockstroh, B. (2019). Environmental adversities and psychotic symptoms: The impact of timing of trauma, abuse, and neglect. Schizophrenia Research, 205, 4–9. 10.1016/j.schres.2017.10.034 29141785

[ref47] Spaccarelli, S., & Kim, S. (1995). Resilience criteria and factors associated with resilience in sexually abused girls. Child Abuse & Neglect, 19(9), 1171–1182. 10.1016/0145-2134(95)00077-L 8528822

[ref48] Steine, I. M., Winje, D., Krystal, J. H., Bjorvatn, B., Milde, A. M., Grønli, J., … Pallesen, S. (2017). Cumulative childhood maltreatment and its dose-response relation with adult symptomatology: Findings in a sample of adult survivors of sexual abuse. Child Abuse & Neglect, 65, 99–111. 10.1016/j.chiabu.2017.01.008 28131947

[ref49] Strobl, C., Boulesteix, A.-L., Zeileis, A., & Hothorn, T. (2007). Bias in random forest variable importance measures: Illustrations, sources and a solution. BMC Bioinformatics, 8(1), 25. 10.1186/1471-2105-8-25 17254353 PMC1796903

[ref50] Svetnik, V., Liaw, A., Tong, C., Culberson, J. C., Sheridan, R. P., & Feuston, B. P. (2003). Random forest: A classification and regression tool for compound classification and QSAR modeling. Journal of Chemical Information and Computer Sciences, 43(6), 1947–1958. 10.1021/ci034160g 14632445

[ref51] Swedo, E. A., Aslam, M. V., Dahlberg, L. L., Niolon, P. H., Guinn, A. S., Simon, T. R., & Mercy, J. A. (2023). Prevalence of adverse childhood experiences among U.S. adults – behavioral risk factor surveillance system, 2011–2020. MMWR. Morbidity and Mortality Weekly Report, 72(26), 707–715. 10.15585/mmwr.mm7226a2 37384554 PMC10328489

[ref52] Taquette, S. R., & Monteiro, D. L. M. (2019). Causes and consequences of adolescent dating violence: A systematic review. Journal of Injury and Violence Research, 11(2), 137. 31263089 10.5249/jivr.v11i2.1061 PMC6646825

[ref53] Teicher, M. H., & Parigger, A. (2015). The ‘Maltreatment and Abuse Chronology of Exposure’ (MACE) scale for the retrospective assessment of abuse and neglect during development. PLoS ONE, 10(2), e0117423. 10.1371/journal.pone.0117423 25714856 PMC4340880

[ref54] Teicher, M. H., Samson, J. A., Polcari, A., & McGreenery, C. E. (2006). Sticks, stones, and hurtful words: Relative effects of various forms of childhood maltreatment. American Journal of Psychiatry, 163(6), 993–1000. 10.1176/ajp.2006.163.6.993 16741199

[ref55] Teicher, M. H., Samson, J. A., Polcari, A., & Andersen, S. L. (2009). Length of time between onset of childhood sexual abuse and emergence of depression in a young adult sample: A retrospective clinical report. The Journal of Clinical Psychiatry, 70(5), 684–691. 10.4088/jcp.08m04235 19358787 PMC4266432

[ref56] Thornberry, T. P., Ireland, T. O., & Smith, C. A. (2001). The importance of timing: The varying impact of childhood and adolescent maltreatment on multiple problem outcomes. Development and Psychopathology, 13(4), 957–979. 10.1017/S0954579401004114 11771916

[ref57] Tomoda, A., Sheu, Y.-S., Rabi, K., Suzuki, H., Navalta, C. P., Polcari, A., & Teicher, M. H. (2011). Exposure to parental verbal abuse is associated with increased gray matter volume in superior temporal gyrus. NeuroImage, 54(Suppl 1), S280–S286. 10.1016/j.neuroimage.2010.05.027 20483374 PMC2950228

[ref58] Tremblay-Perreault, A., & Hébert, M. (2020). Uncovering the associations between child sexual abuse, peer victimization and behavior problems using child, parent and teacher reports. Journal of School Violence, 19(3), 336–348. 10.1080/15388220.2019.1697276

[ref59] Trickett, P. K., Mennen, F. E., Kim, K., & Sang, J. (2009). Emotional abuse in a sample of multiply maltreated, urban young adolescents: Issues of definition and identification. Child Abuse & Neglect, 33(1), 27–35. 10.1016/j.chiabu.2008.12.003 19178945 PMC5576987

[ref60] Widom, C. S., DuMont, K., & Czaja, S. J. (2007). A prospective investigation of major depressive disorder and comorbidity in abused and neglected children grown up. Archives of General Psychiatry, 64(1), 49–56. 10.1001/archpsyc.64.1.49 17199054

[ref61] Wiersma, J. E., Hovens, J. G. F. M., Van Oppen, P., Giltay, E. J., Van Schaik, D. J. F., Beekman, A. T. F., & Penninx, B. W. J. H. (2009). The importance of childhood trauma and childhood life events for chronicity of depression in adults. The Journal of Clinical Psychiatry, 70(7), 983–989. 10.4088/JCP.08m04521 19653975

[ref62] Yancey, C. T., & Hansen, D. J. (2010). Relationship of personal, familial, and abuse-specific factors with outcome following childhood sexual abuse. Aggression and Violent Behavior, 15(6), 410–421. 10.1016/j.avb.2010.07.003

[ref63] Zhu, J., Lowen, S. B., Anderson, C. M., Ohashi, K., Khan, A., & Teicher, M. H. (2019). Association of prepubertal and postpubertal exposure to childhood maltreatment with adult amygdala function. JAMA Psychiatry, 76(8), 843. 10.1001/jamapsychiatry.2019.0931 31241756 PMC6596335

[ref64] Zlotnick, C., Mattia, J., & Zimmerman, M. (2001). Clinical features of survivors of sexual abuse with major depression. Child Abuse and Neglect, 25(3), 357–367. 10.1016/s0145-2134(00)00251-9. 11414395

