# Supplementary material for: Childhood sexual abuse and lifetime depressive symptoms: the importance of type and timing of childhood emotional maltreatment
Source: Psychol Med. 2024 Dec 2;54(15):4385–95. doi: 10.1017/S003329172400268X (PMC11650171; doi:10.1017/S003329172400268X)
Supplement: Hutson et al. supplementary material 1 — Hutson et al. supplementary material [file S003329172400268Xsup001.docx]

**Childhood sexual abuse and lifetime depression: The importance of type and timing of childhood emotional maltreatment**

***Supplemental Information***

**Supplemental Methods**

- Parent study inclusion/exclusion criteria
- Study enrollment flow
- Assessment of childhood sexual abuse

**Supplemental Results**

- Sexual abuse characteristics
- Significant pairwise comparisons of emotional maltreatment severity

**Supplemental Methods**

**Inclusion/exclusion criteria**

Participants were recruited for a parent neuroimaging study investigating early life stress and depression at the Center for Depression, Anxiety and Stress Research (CDASR) at McLean Hospital (Early Life Stress and Depression: Molecular and Functional Imaging Approaches; Protocol 2020P001470). The larger neuroimaging study from which the present study’s data were collected enrolled only women due to expected sex-specific differences in: (i) abuse-related variables (e.g., child-perpetrator gender), (ii) variables unrelated to abuse (e.g., brain development; Pechtel et al., 2014), and (iii) HPA axis reactivity (Young & Korszun, 2010). Recruitment of participants was done mainly using advertisements on internet sites, including: Craigslist, Reddit, Google, Meta, Partners RALLY system, SONA, Boston-area student job board and research sites, job recruitment sites, and Research Match. Additional recruitment methods included flyers and outreach to participants from past studies who consented to be re-contacted. Participants were screened using a self-report recruitment survey that included the Beck Depression Inventory (BDI-II) (Beck et al., 1996) followed by a phone screen conducted by trained research assistants. Participants who were eligible to continue following this phone screen completed the Maltreatment and Abuse Chronology of Exposure (MACE) (Teicher & Parigger, 2015) to assess a detailed maltreatment history. Detailed inclusion and exclusion criteria are found below.

**Inclusion criteria for all subjects:**

- Females of all races and ethnic origins
- Ages 20-32 years old
- Right-handed
- Capable of providing written informed consent
- No psychotropic medication at the time of enrollment and have not taken any psychotropic medications for at least 2 weeks (6 weeks for fluoxetine; 6 months for neuroleptics and dopaminergic drugs (e.g., bupropion, Adderall); 2 weeks for benzodiazepines; and 2 weeks for any other antidepressants.)
- Normal or corrected-to-normal vision and hearing
- Fluency in written and spoken English
- Absence of first-degree relatives with a history of a psychotic disorder or psychotic symptoms

**Additional inclusion criteria for women with current depressive symptoms and CSA between ages 11-18 (MDD/CSA group):**

- First instance of contact sexual abuse between ages 11-18 (ascertained using the MACE).
- A score of at least 14 on the BDI-II.

**Additional inclusion criteria for women with remitted depressive symptoms and CSA between ages 11-18 (rMDD/CSA group):**

- First instance of contact sexual abuse between ages 11-18 (ascertained using the MACE).
- At least one major depressive episode within the past 5 years that has been in remission for at least 2 months, ascertained through a phone interview.
- A score less than 14 on the BDI-II.

**Additional inclusion criteria for women with current depressive symptoms and no history of sexual abuse (MDD/No CSA group):**

- No history of sexual abuse at any age.
- A score of at least 14 on the BDI-II.

**Exclusion criteria for all subjects:**

- Participants with suicidal ideation if participation was deemed unsafe by the study clinician (these participants would be immediately referred to appropriate clinical treatment).
- Failure to meet MRI safety requirements.
- Substance and drug use exclusions:
- More than 15 lifetime alcohol-induced blackouts
- History of regular use (5-7 times/week) of marijuana before the age of 15
- Lifetime history of other recreational drug use beyond the following limits, and/or last use within the past 3 months
  - 10 uses of mushrooms
  - 5 uses of anxiolytics, other hallucinogens (i.e., LSD, Ecstasy), opioids, or stimulants (not including prescribed stimulants such as methylphenidate). Prescribed opioids for a limited period (e.g., post-surgery) were allowed outside of the past 3 months.
  - 1 use of inhalants, IV drugs, crack cocaine, or crystal methamphetamine.
- Use of drug or herbal supplement for depression or those that could affect stress response within the past 3 weeks.
- Use of any medication that affects blood flow or blood pressure within the past 3 weeks.
- Use of metformin within the past 6 months.
- Medical exclusions:
  - Serious or unstable medical illness
  - Hemophilia
  - Diabetes with poor glucose control
  - History of chronic migraines (>15 days/month)
  - History of seizure disorder
  - Any history of significant head injury or concussion with loss of consciousness for two minutes or more, or head injury with present lingering functional or psychological impact.
  - History of electroconvulsive therapy (ECT).
- Comorbidity exclusions:
- Past or current DSM-V diagnosis of: obsessive compulsive disorder, schizophrenia, schizoaffective disorder, delusional disorder, psychotic disorders not otherwise specified, bipolar disorder, patients with mood congruent or mood incongruent psychotic features, autism or any other pervasive developmental disorder, organic mental disorder, or anorexia. A history of bulimia was allowed if it had been in remission for at least 3 months. A history of post-traumatic stress disorder was allowed if the symptoms had been subthreshold for the past two years.
- History of moderate or severe substance or alcohol use disorder within the past 6 months; or mild substance or alcohol use disorder within the last 6 months (with the exception of cocaine or stimulant abuse, which will lead to automatic exclusion).

**Study enrollment flow**


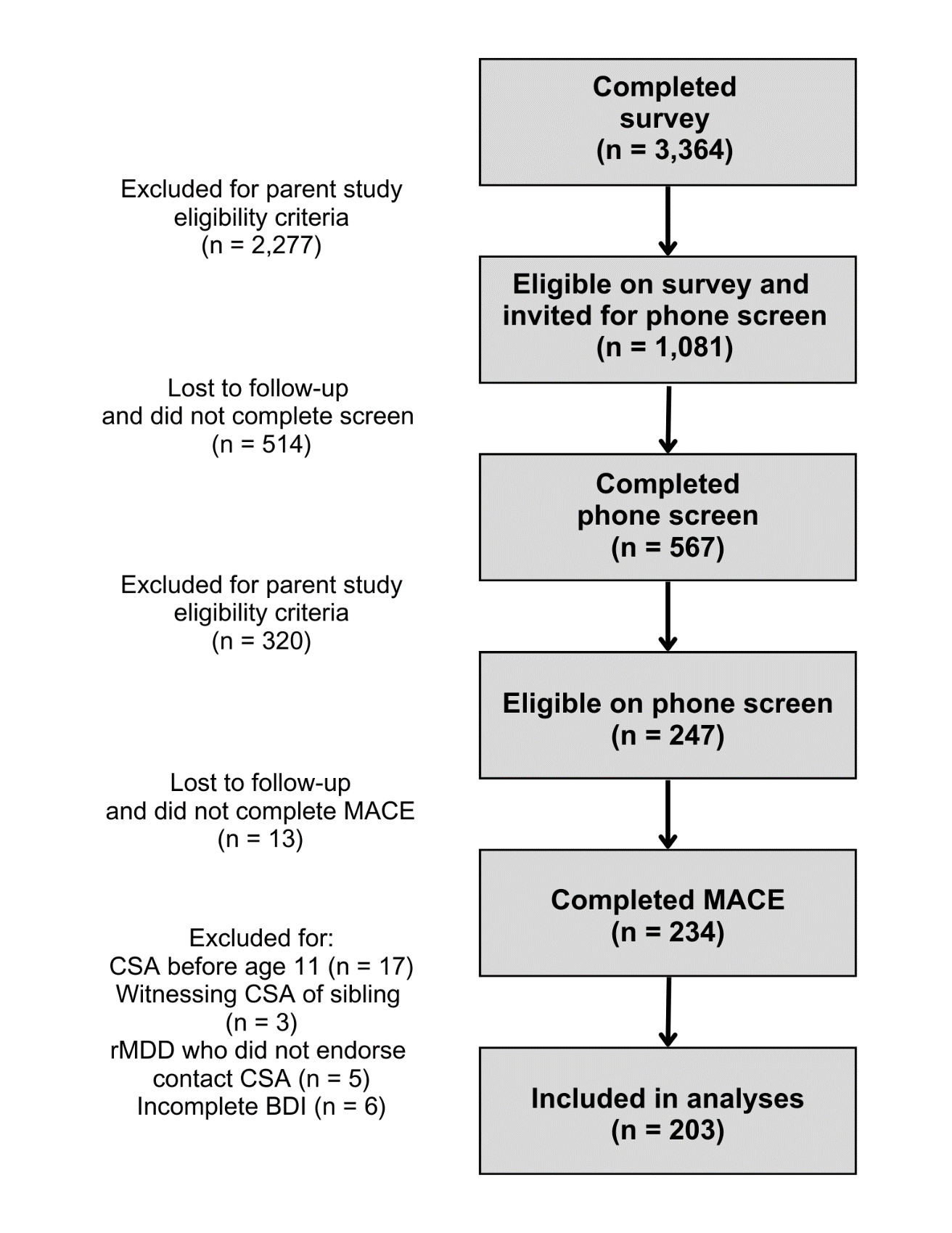


**Assessment of childhood sexual abuse**

The assessment of sexual abuse was done using the 75-item MACE-X (Teicher & Parigger, 2015). The original sexual abuse category of the MACE includes 8 items, with significant exposure defined as the endorsement of 2 or more questions. However, there are 4 additional questions that assess sexual abuse which are not scored or included in the sexual abuse category. For the purpose of the current study, a revised measure of contact sexual abuse was created to capture all items that describe contact sexual abuse. Our revised sexual abuse category included 10 MACE items, with the endorsement of any one of them constituting significant sexual abuse. The table below indicates which questions contribute to the sexual abuse category as originally defined on the MACE and which contributed to our revised sexual abuse measure.

| MACE-X Questions | Sexual Abuse (Teicher & Parigger, 2015) | Contact Sexual Abuse (Present Study) |
| --- | --- | --- |
| Parents, stepparents, or other adults living in the house | | |
| Made inappropriate sexual comments or suggestions to you. | ✓ |  |
| Touched or fondled your body in a sexual way. | ✓ | ✓ |
| Had you touch their body in a sexual way. | ✓ | ✓ |
| Attempted to have any type of sexual intercourse (oral, anal, or vaginal) with you. |  | ✓ |
| Actually had any type of sexual intercourse (oral, anal or vaginal) with you. |  | ✓ |
| Adults or older individuals not living in the house | | |
| Made inappropriate sexual comments or suggestions to you. |  |  |
| Touched or fondled your body in a sexual way. | ✓ | ✓ |
| Had you touch their body in a sexual way. |  | ✓ |
| Attempted to have any type of sexual intercourse (oral, anal, or vaginal) with you. |  | ✓ |
| Actually had any type of sexual intercourse (oral, anal or vaginal) with you. | ✓ | ✓ |
| Children your own age or older | | |
| Forced you to engage in sexual activity against your will. | ✓ | ✓ |
| Forced you to do things sexually that you did not want to do. | ✓ | ✓ |

**Supplemental Results**

**Supplemental Table 1. Sexual abuse characteristics**

|  | **MDD/CSA**  **(n = 69)** | **rMDD/CSA**  **(n = 21)** | **Total**  **(n = 90)** |  |  |
| --- | --- | --- | --- | --- | --- |
| **Perpetrator Relationship** |  |  |  | ***X^2^*** | ***p*** |
| Adult living in the home, *n* (%) | 10 (14) | 0 (0) | 10 (11) | 3.42 | .064 |
| Adult living outside the home, *n* (%) | 37 (54) | 14 (67) | 51 (57) | 1.12 | .291 |
| Peer their own age or older, *n* (%) | 47 (68) | 8 (38) | 55 (61) | 6.11 | .013 |
|  |  |  |  | ***t*** | ***p*** |
| **Number of CSA perpetrator categories endorsed (avg ± SD)** | 1.38 ± 0.60 | 1.05 ± 0.22 | 1.30 ± 0.55 | 3.82 | < .001 |
|  |  |  |  | ***t*** | ***p*** |
| **Number of years at which CSA was endorsed (avg ± SD)** | 2.51 ± 1.62 | 1.86 ± 1.01 | 2.36 ± 1.52 | 1.73 | .087 |
| **Ages of CSA** |  |  |  |  |  |
| Age 11, *n* (%) | 4 (6) | 1 (5) | 5 (6) |  |  |
| Age 12, *n* (%) | 7 (10) | 3 (14) | 10 (11) |  |  |
| Age 13, *n* (%) | 19 (28) | 2 (10) | 21 (23) |  |  |
| Age 14, *n* (%) | 16 (23) | 5 (24) | 21 (23) |  |  |
| Age 15, *n* (%) | 21 (30) | 5 (24) | 26 (29) |  |  |
| Age 16, *n* (%) | 35 (51) | 9 (43) | 44 (49) |  |  |
| Age 17, *n* (%) | 39 (57) | 7 (33) | 46 (51) |  |  |
| Age 18, *n* (%) | 32 (46) | 7 (33) | 39 (43) |  |  |

**Supplemental Table 2. Significant pairwise comparisons of emotional maltreatment severity.**

| **Age** (years) | **Pairwise Comparison** | | ***p*** | **95% CI** | |
| --- | --- | --- | --- | --- | --- |
|  |  |  |  | **Lower Bound** | **Upper Bound** |
| 3 | MDD/CSA | rMDD/CSA | .025* | .247 | 4.879 |
| 4 | MDD/CSA | rMDD/CSA | .030* | .231 | 6.134 |
| 5 | MDD/CSA | rMDD/CSA | .030* | .274 | 7.437 |
| 6 | MDD/CSA | rMDD/CSA | .042* | .115 | 8.137 |
| 8 | MDD/CSA | rMDD/CSA | .033* | .298 | 9.316 |
| 10 | MDD/CSA | rMDD/CSA | .013* | 1.003 | 11.100 |
| 11 | MDD/CSA | rMDD/CSA | .006** | 1.644 | 12.249 |
| 12 | MDD/CSA | rMDD/CSA | < .001*** | 2.813 | 13.122 |
|  | MDD/CSA | MDD/No CSA | .009** | .785 | 7.164 |
| 13 | MDD/CSA | rMDD/CSA | < .001*** | 3.060 | 13.147 |
|  | MDD/CSA | MDD/No CSA | .003** | 1.182 | 7.424 |
| 14 | MDD/CSA | rMDD/CSA | < .001*** | 3.517 | 13.169 |
|  | MDD/CSA | MDD/No CSA | < .001*** | 2.214 | 8.187 |
| 15 | MDD/CSA | rMDD/CSA | < .001*** | 4.081 | 13.592 |
|  | MDD/CSA | MDD/No CSA | < .001*** | 2.827 | 8.713 |
| 16 | MDD/CSA | rMDD/CSA | < .001*** | 4.633 | 14.080 |
|  | MDD/CSA | MDD/No CSA | < .001*** | 3.018 | 8.863 |
| 17 | MDD/CSA | rMDD/CSA | < .001*** | 4.369 | 13.722 |
|  | MDD/CSA | MDD/No CSA | < .001*** | 2.146 | 7.949 |
| 18 | MDD/CSA | rMDD/CSA | < .001*** | 2.568 | 12.125 |
|  | MDD/CSA | MDD/No CSA | .017* | .476 | 6.390 |

Sidak post-hoc corrections were applied for each comparison. Significance: * *p* < .05; ** *p* < .01; *** *p* < .001.

**References**

Beck, A. T., Steer, R. A., & Brown, G. (1996). *Beck Depression Inventory–II*. APA PsycTests. https://doi.org/10.1037/t00742-000

Pechtel, P., Lyons-Ruth, K., Anderson, C. M., & Teicher, M. H. (2014). Sensitive periods of amygdala development: The role of maltreatment in preadolescence. *NeuroImage*, *97*, 236–244. https://doi.org/10.1016/j.neuroimage.2014.04.025

Teicher, M. H., & Parigger, A. (2015). The ‘Maltreatment and Abuse Chronology of Exposure’ (MACE) Scale for the Retrospective Assessment of Abuse and Neglect During Development. *PLOS ONE*, *10*(2), e0117423. https://doi.org/10.1371/journal.pone.0117423

Young, E., & Korszun, A. (2010). Sex, trauma, stress hormones and depression. *Molecular Psychiatry*, *15*(1), 23–28. https://doi.org/10.1038/mp.2009.94
